# The determinants of sustained adherence to COVID-19 preventive measures among older Syrian refugees in Lebanon

**DOI:** 10.1371/journal.pone.0268851

**Published:** 2023-06-22

**Authors:** Nisreen Salti, Stephen J. McCall, Berthe Abi Zeid, Noura El Salibi, Marwan Alawieh, Zeinab Ramadan, Hala Ghattas, Sawsan Abdulrahim

**Affiliations:** 1 Department of Economics, American University of Beirut, Beirut, Lebanon; 2 Center for Research on Population and Health, Faculty of Health Sciences, American University of Beirut, Beirut, Lebanon; 3 Norwegian Refugee Council, Beirut, Lebanon; 4 Department of Health Promotion and Community Health, Faculty of Health Sciences, American University of Beirut, Beirut, Lebanon; Universitas Syiah Kuala, INDONESIA

## Abstract

**Introduction:**

Lebanon has battled the COVID-19 pandemic in the midst of an economic crisis. The evolution of the pandemic and a fragile health system have meant that public health policy has had to rely heavily on non-pharmaceutical interventions for disease control. However, changes in disease dynamics, an unraveling economy, and pandemic fatigue have meant that disease control policies need to be updated.

**Methods:**

Using recent and timely data on older (50 years and above) Syrian refugees in Lebanon, this paper uses multivariate linear probability models to explore the determinants of adherence to two non-pharmaceutical COVID-19 prevention measures (wearing a mask and avoiding social gatherings) among this high-risk subgroup in a vulnerable population. Among respondents who report adhering to these measures, the paper also investigates the determinants of sustained adherence over a period of 6 months.

**Results:**

The findings suggest that no individual-level characteristics were robustly associated with mask wearing. For avoiding social gatherings, education was inversely associated with adherence to this preventive measure. Avoiding social gatherings was also significantly lower for residents of informal tented settlements (ITSs). Among initial adherents, and for both preventive practices, ITS dwellers were also significantly less likely to maintain adherence.

**Conclusion:**

Identifying variables associated with adherence to non-pharmaceutical preventive practices, particularly for vulnerable groups, can help inform and refine interventions in the face of changing conditions. The material, physical, administrative and socio-economic constraints of life in an ITS suggest that avoiding social gatherings is hardly feasible. Yet despite the challenging conditions of ITSs, the indication to wear a mask is initially complied with, suggesting that tailoring policies to the limits and constrains of context can lead to successful outcomes even in very adverse settings.

## Introduction

Lebanon is suffering a confluence of crises: an economic and financial collapse that started in 2019 and has slashed Gross Domestic Product (GDP), doubled the poverty rate, and mired the country in hyperinflation; the COVID-19 pandemic since early 2020; and the explosion at the Port of Beirut in August of 2020 that caused extensive human and material damage. As a result, the country’s healthcare system has been pushed to the brink of collapse [1–3].

The emergence of new variants of the coronavirus, the ensuing surges In recorded cases, as well as the slow rollout of vaccination campaigns have meant that Lebanon, like many other countries, continues to rely on non-pharmaceutical interventions (NPIs) to curb the spread of the COVID-19 pandemic, until herd immunity is reached [4–6]. In fact, with Lebanon’s economy already in freefall and its public health infrastructure severely compromised, low cost NPIs can play a pivotal role in preventing and controlling the spread of the virus. Research suggests that mask wearing and reducing contact with others are effective NPIs for preventing infection from COVID-19 [7–10]. However, the pandemic is about to enter its third year, so continued adherence to some of these preventive measures, such as staying at home except for essential outings, restricting travel, and limiting visitors, is proving increasingly difficult to maintain. Indeed, ‘pandemic fatigue’ has meant that even adherence to far less costly preventive measures, such as mask wearing, is on the wane.

Continued reliance on NPIs implies that the potential effects of ‘pandemic fatigue’ on adherence should be of concern, as the World Health Organization (WHO) was already warning in June of 2020 [11]. While evidence of pandemic fatigue setting in is ample at a global scale [12, 13], it should be all the more relevant in a context like Lebanon, in which an economic crisis has made daily life increasingly challenging and has largely depleted people’s motivation to comply with new behavioral recommendations. Among the most exposed to both the economic crisis and the public health emergency in Lebanon are Syrian refugees [14]. Beyond their socio-economic vulnerability even prior to the economic crisis [15, 16], Syrian refugees are also more vulnerable to COVID-19 because they tend to live in crowded conditions with limited access to sanitation and public health infrastructure [17]. Among Syrian refugees, older adults are particularly exposed to the risk of severe morbidity and death from COVID-19 [18–20].

The aim of the paper is to use recent and timely data on older Syrian refugees (ages 50+) to explore the individual- and household-level determinants of adherence to wearing a mask and avoiding social gatherings (such as weddings and funerals) among this high-risk subgroup in a vulnerable population, as well as the determinants of sustained adherence over a period of 6 months among those who report to abide by these recommendations. The focus is on these two practices since they are among the lowest cost, effective COVID-19 prevention NPIs.

Identifying the factors associated with these two NPIs may be of particular policy value because in the face of the enormous economic and logistical challenges that Lebanon is mired with today, public health policy makers in Lebanon today have very limited resources for curbing the spread of COVID-19. Similarly, identifying factors associated with waning adoption of these measures by those most at risk among vulnerable populations could alleviate some of the pressure on an already frail public health infrastructure. This exercise could also help in fine-tuning messaging for interventions such as mobility restrictions and vaccination in the face of pandemic fatigue.

A growing literature has emerged on the correlates and factors associated with the use of masks and the adoption of other preventive measures globally [21, 22]. The literature is also rich with country studies examining the predictors and correlates of mask wearing and other preventive measures. Daoust identified the correlates of compliance with preventive measures across 27 countries, with a focus on older adults [23]. In the context of Lebanon, some work has tried to identify correlates of adherence to preventive measures [24], sometimes for some subpopulations [25–27], while other work has looked at the role of specific interventions in affecting or mediating adherence [28].

While the identification of such correlates may have been informative for policy makers trying to devise prevention and control policies, COVID-19 control strategies have to reckon with the fact that people’s behavior also changes over time. Changing behavior means that subgroups that were initially adherent may not remain so for long. Pandemic response policies need to be updated accordingly. However, research that aims to shed light on the dynamics of adherence over time is more scant.

Chan et al. use repeated cross-sectional data to examine the socio-economic and demographic correlates of changing adherence to social distancing and personal hygiene measures in Hong Kong during the first and third waves of COVID-19 [29]. They find improved adherence to mask wearing and decreasing compliance with social distancing measures (including avoidance of gatherings, of public places, and of international travel). This is in line with evidence from multi-country studies which suggests that adherence to sensitizing behaviors such as physical distancing has decreased, while adherence to habituating behaviors such as mask wearing has increased [13]. Like Chan et al., Petherick et al. also base their results on observational data, but their repeated cross sections are more frequent and span 14 countries [13, 29].

Unlike both Chan et al. and Petherick et al., we do not seek to measure cross-population changes in compliance over time. Instead, we focus on one subgroup (older adults) in one subpopulation (Syrian refugees), following the same individuals over time, and tracking their self-reported compliance behavior on two occasions that are 6 months apart. The timing of our sample means that unlike Chan et al., we are not limited to observations only during periods of surges of COVID-19 cases [13, 29].

Research on the dynamics of compliance in Lebanon is still very preliminary: Makki et al [30] document some evidence in support of pandemic fatigue in Lebanon, but their findings are based on a very small sample (n = 30, only some of whom are from Lebanon) from a pilot study. With their sample size, they are unable to identify the correlates of behavioral fatigue in adherence to preventive measures.

This paper shares some of the aims of Daoust [23], but rather than only profiling compliers by using cross-sectional data to identifying correlates of adherence to a preventive measure, we also attempt to identify individual-level correlates of compliers who maintain compliance over 6 months vs. those who give up on it. If uncovering the correlates of adherence to preventive measures was informative for COVID-19 response policies early on during the pandemic, identifying the characteristics associated with waning compliance is of relevance to updating these policies, fine-tuning the target populations, and sharpening the messaging in response to ‘pandemic fatigue’.

## Materials and methods

### Study design

#### Sampling frame and sample selection

The analysis used data from a study which aimed to examine the vulnerabilities of older Syrian refugees living in Lebanon during the COVID-19 pandemic. The study population included all households with at least one adult known to be 50 years or older who benefited from the services offered by the Norwegian Refugee Council (NRC) between 2017 and 2020. All these households (n = 17,384) were contacted and a member 50 or older was invited to participate to the study. If there were more than one Syrian refugee aged 50 years or older, one participant was chosen randomly. Participants aged 65 years or older were assessed for capacity to consent using five modified items from the University of California, San Diego Brief Assessment of Capacity to Consent [31]. Eligible respondents who provided oral consent (n = 3,838) were then contacted to complete five different telephone surveys over 8 months. This paper used data from the first (n = 3,838) and fourth (n = 2,991) waves of interviews (October-December of 2020 and February-May 2021, respectively). The data collection exercise was approved by the Social and Behavioral Sciences Institutional Review Board of the American University of Beirut (Reference: SBS-2020-0329). Data collectors were trained to make every effort to protect participants’ autonomy. The data collected through the phone survey was anonymized.

#### Survey instrument and variables

The survey was designed by a team including academics, humanitarian actors, local government officials and focal points from the community of Syrian refugees. The questionnaires were developed based on validated modules with additional questions specific to the context and research objectives and priorities identified by the community. The questionnaires contained different modules in each wave, include information on demographic characteristics of the individual and their household, ownership of assets, labor market status, health status, the receipt of cash assistance, including COVID-19 related cash assistance, COVID-related behaviors, food and water insecurity, decision making and social support, exposure to violence and security risks. Survey interviews were conducted in Arabic over the phone by trained data collectors, and data was entered into Kobo Toolbox. Full questionnaires are available on ScholarWorks [32]. Details about the study design and the development of the study tool were published elsewhere [33].

Respondents were asked on 2 occasions, close to 6 months apart, about their adherence to two different practices related to recommended preventive measures. The recommended practices are: (i) wearing a mask and (ii) avoiding social occasions (such as weddings and funerals). The first is a habituating behavior, the second a sensitizing one, but they are both associated with relatively lower costs on the complier when compared to the other NPIs for COVID-19 control such as sheltering in place and limiting travel and movement.

### Statistical analysis

Linear probability models were used to determine the correlates of adherence to each of the two preventive behaviors separately using the entire sample, and determinants of sustained adherence 6 months later in the subsample of those who initially report adherence. This estimation procedure was chosen for the simplicity of interpretation of the results. Linear probability models used the discrete adherence behavior variables as dependent variables and investigated partial correlations with some of the associated socio-demographic, labor market, health and household-level variables. Individual-level variables included socio-demographic characteristics (sex, age, marital status, education), labor market status (employment status), health (smoker status, chronic conditions), self-reported susceptibility to COVID-19, a variable measuring the decision-making process in the household about adherence to preventive behaviors. Household-level variables included household size, socio-economic variables, governorate of residence, an indicator of residence in an ITS, and an indicator measuring receipt of COVID-19 cash assistance. Socio-economic status was measured using food security status (the Food Insecurity Experience Scale) and asset ownership (a variable counting the number of affirmative answers to questions about the ownership of the following 15 assets: car, motorbike/scooter, van/pickup truck, bicycle, gas stove, oven, refrigerator, iron, heater, water heater, washing machine, TV, computer, mobile phone, access to the internet). All regressions also included variables for the week of data collection. The inclusion of fixed effects for week of data collection allows us to control for any macro-level effects that prevail for the entire population in that week, including the economic environment, any policies that are in place, or any other ambient conditions that might have affected the whole sample at the time.

Regressions of sustained adherence also included additional dynamic variables measuring the respondent’s COVID-19 history, changes in their labor market status, and changes in their type of shelter. We confirmed the robustness of the findings by re-running the basic regressions as probit models, assuming normally distributed disturbances.

## Results and discussion

### Characteristics on respondents

Summary statistics for the data used in the regressions are presented in Tables 1 and 2. Table 1 shows the mean (proportion for categorical variables) and standard deviation of the variables included in the regressions for mask wearing in Panel A and for avoiding social gatherings in Panel B. Individual-level characteristics included demographic information (sex, age, marital status), education, labor market status, an indicator of whether the COVID-19 preventive behaviors were the respondent’s own decision, an indicator of the respondent’s reported susceptibility of COVID-19, smoker status, and an indicator for the respondent having a chronic health condition. Household-level characteristics included household size, assets, an indicator for food insecurity, an indicator for receiving cash assistance, an indicator for living in an Informal Tented Settlement (ITS), in addition to district of residence.

**Table 1:** Summary statistics.

Panel A: Subsample used in regressions of mask wearing (N = 986)		
**Variables**	**Mean or proportion (%)**	**(SD)**
**Individual Level**		
**Female**	27.7	(44.8)
**Married**	82.6	(37.8)
**Widowed**	14.0	(34.7)
**Age**	57.12	(6.93)
**Education**		
Never attended school	42.0	(49.4)
Elementary school	31.1	(46.3)
Preparatory school	17.1	(37.7)
Secondary school and above	9.8	(29.7)
**Employed**	14.7	(35.4)
**Own decision**	35.7	(47.9)
**Current smoker**	75.3	(43.1)
**Susceptible**	74.6	(43.5)
**Chronic condition**	64.5	(47.9)
**Household level**		
**Household size**	7.99	(4.01)
**Assets**	5.66	(1.40)
**Food insecurity**	91.8	(27.5)
**Cash assistance**	70.3	(45.7)
**Living in ITS**	65.3	(47.6)
**Governorate**		
Akkar	14.1	(34.8)
Baalbeck-Hermel	14.2	(34.9)
Beirut	1.3	(11.4)
Beqaa	43.1	(49.5)
Mount Lebanon	0.3	(5.5)
Nabatieh	3.6	(18.8)
North	14.8	(35.5)
South	8.5	(27.9)
**Adherence measures**		
**Wears mask**	83.4	(37.2)
Panel B: Subsample used in regressions of avoiding social gatherings (N = 1,571)		
**Variables**	**Mean or proportion (%)**	**(SD)**
**Individual Level**		
**Female**	26.0	(43.9)
**Married**	81.9	(38.5)
**Widowed**	14.9	(35.8)
**Age**	57.3	(6.87)
**Education**		
Never attended school	38.8	(48.7)
Elementary school	30.4	(46.0)
Preparatory school	19.9	(39.9)
Secondary school and above	11.9	(31.1)
**Employed**	14.8	(35.5)
**Own decision**	33.9	(47.3)
**Current smoker**	76.8	(42.2)
**Susceptible**	72.2	(44.8)
**Chronic condition**	64.2	(48.0)
**Household level**		
**Household size**	7.97	(3.92)
**Assets**	5.81	(1.37)
**Food insecurity**	91.3	(28.1)
**Cash assistance**	70.7	(45.5)
**Living in ITS**	41.0	(49.2)
**Governorate**		
Akkar	17.0	(37.6)
Baalbeck-Hermel	10.9	(31.2)
Beirut	1.1	(10.6)
Beqaa	36.7	(48.2)
Mount Lebanon	0.4	(6.66)
Nabatieh	5.0	(21.9)
North	17.4	(37.9)
South	11.3	(31.7)
**Adherence measures**		
**Avoid social gatherings**	92.0	(27.1)

**Table 2:** Summary statistics for subsample of initial adherents (N = 675).

Variables	Mean or proportion (%)	(SD)
**Sustained mask wearing**	74.67	(43.5)
**Sustained avoiding social gatherings**	88.6	(31.8)
**Female**	27.2	(44.6)
**Married**	85.0	(35.7)
**Widowed**	11.8	(32.3)
**Age**	56.9	(6.46)
**Education**		
Never attended school	41.0	(49.2)
Elementary school	31.5	(46.5)
Preparatory school	17.8	(38.2)
Secondary school and above	9.7	(29.5)
**Employed**	9.78	(29.7)
**Job loss (due to COVID-19)**	20.1	(40.1)
**Salary reduction (due to COVID-19)**	21.5	(41.1)
**Own decision**	35.4	(47.8)
**Current smoker**	76.6	(42.4)
**Chronic condition**	63.0	(48.3)
**Susceptible**	76.1	(42.6)
**Had COVID-19**	16.2	(12.7)
**Household level**		
**Household size**	8.0	(3.86)
**Assets**	5.72	(1.38)
**Severe food insecurity**	36.3	(48.1)
**Cash assistance**	80.3	(39.8)
**Living in ITS**	55.2	(49.7)
**Moved to ITS**	12.0	(32.5)
**Moved from ITS**	13.0	(33.7)
**Governorate**		
Akkar	15.1	(35.8)
Baalbeck-Hermel	14.4	(35.1)
Beirut	0.3	(5.44)
Beqaa	42.1	(49.4)
Mount Lebanon	1.2	(10.8)
Nabatieh	4.6	(20.9)
North	12.7	(33.4)
South	9.6	(29.5)
**Month (in 2021)**		
February	14.5	(35.2)
March	51.1	(50.0)
April	14.8	(35.5)
May	19.6	(39.7)
**Duration between interviews**		
<150 days	27.0	(44.4)
150–159 days	66.4	(21.6)
160–169 days	4.9	(21.6)
170+ days	1.7	(13.2)

Table 2 reports summary statistics for variables included in the regressions for sustained adherence. So Table 2 only shows data on initial adherents. In addition to the variables in Table 1, these also included variables measuring the respondent’s COVID-19 history, any reported changes in their labor market status and earnings, and measures of respondents moving into or out of ITSs since the first wave of data collection, as well as the length of time elapsed between the interviews for the two waves.

### Results for mask wearing

#### Adherence to mask wearing

Table 3 shows the results of regressions for mask wearing.

**Table 3:** Adherence to mask wearing.

	Regression 1		Regression 2		Regression 3	
	β	(S.E.)	β	(S.E.)	β	(S.E.)
	[95% CI]		[95% CI]		[95% CI]	
**Individual-level variables**						
Female	0.03	(0.03)	0.03	(0.03)	0.03	(0.03)
	[-0.04:0.10]		[-0.04:0.10]		[-0.03:0.09]	
Married	0.11	(0.07)	0.11	(0.07)	0.13	(0.08)
	[-0.03:0.25]		[-0.02:0.24]		[-0.03:0.29]	
Widowed	0.03	(0.07)	0.03	(0.07)	0.03	(0.06)
	[-0.11:0.18]		[-0.11:0.17]		[-0.08:0.15]	
Age	-0.003**	(0.002)	-0.002	(0.002)	-0.002	(0.002)
	[-0.006:0.000]		[-0.01:0.001]		[-0.01:0.001]	
Education						
Never attended school	Ref		Ref		Ref	
Elementary school	0.02	(0.03)	0.02	(0.03)	0.02	(0.03)
	[-0.04:0.08]		[-0.04:0.08]		[-0.03:0.08]	
Preparatory school	0.02	(0.03)	0.01	(0.04)	0.01	(0.04)
	[-0.04:0.08]		[-0.06:0.09]		[-0.05:0.08]	
Secondary school +	0.05	(0.04)	0.05	(0.04)	0.05	(0.04)
	[-0.04:0.14]		[-0.04:0.14]		[-0.02:0.12]	
Employed	0.03	(0.03)	0.03	(0.03)	0.03	(0.03)
	[-0.04:0.09]		[-0.04:0.09]		[-0.03:0.1]	
Susceptible			0.005	(0.03)		
			[-0.05:0.06]			
Own decision			0.03	(0.02)		
			[-0.02:0.08]			
Smoker	0.01	(0.03)	0.008	(0.03)	0.01	(0.03)
	[-0.04:0.07]		[-0.05:0.06]		[-0.04:0.07]	
Chronic condition	0.007	(0.02)	0.007	(0.02)	0.01	(0.02)
	[-0.04:0.06]		[-0.01:0.02]		[-0.04:0.06]	
**Household-level variables**				** **		
Household size	-0.005	(0.003)	-0.005	(0.003)	-0.005	(0.003)
	[-0.01:0.001]		[-0.01:0.001]		[-0.01:0.001]	
Assets	0.003	(0.01)	0.003	(0.01)	0.003	(0.01)
	[-0.04:0.07]		[-0.01:0.02]		[-0.01:0.02]	
Food insecure	0.04	(0.04)	0.03	(0.04)	0.04	(0.05)
	[-0.05:0.12]		[-0.05:0.12]		[-0.05:0.13]	
Cash assistance	0.01	(0.03)	0.01	(0.03)	0.02	(0.03)
	[-0.04:0.07]		[-0.04:0.07]		[-0.04:0.07]	
ITS	-0.01	(0.03)	-0.01	(0.03)	-0.01	(0.03)
	[-0.07:0.06]		[-0.07:0.06]		[-0.07:0.06]	
**Governorate**				** **		
Akkar	Ref		Ref		Ref	
Baalbeck-Hermel	0.01	(0.04)	0.01	(0.04)	0.01	(0.04)
	[-0.08:0.10]		[-0.08:0.1]		[-0.07:0.1]	
Beirut	0.08	(0.11)	0.08	(0.11)	0.07	(0.08)
	[-0.14:0.29]		[-0.13:0.3]		[-0.08:0.22]	
Beqaa	0.01	(0.04)	0.01	(0.04)	0.01	(0.04)
	[-0.07:0.08]		[-0.07:0.08]		[-0.06:0.08]	
Mount Lebanon	-0.21	(0.22)	-0.21	(0.22)	-0.24	(0.31)
	[-0.64:0.21]		[-0.64:0.21]		[-0.84:0.37]	
Nabatieh	-0.03	(0.07)	-0.03	(0.07)	-0.02	(0.08)
	[-0.17:0.11]		[-0.17:0.11]		[-0.17:0.12]	
North	-0.03	(0.04)	-0.03	(0.04)	-0.03	(0.05)
	[-0.12:0.05]		[-0.12:0.05]		[-0.13:0.06]	
South	0.089^+^	(0.05)	0.09^+^	(0.05)	0.10*	(0.03)
	[-0.02:0.19]		[-0.01:0.19]		[0.03:0.17]	
	**R** ^**2**^	0.06	**R** ^**2**^	0.06	**Pseudo- R** ^**2**^	0.07
	**F(34, 951)**	1.80	**F(36,949)**	1.74	**Chi2(34)**	61.16
	**Observations**			986		986

+ p<0.1

* p<0.05

** p<0.01. All regressions include fixed effects for the week of data collection.

Regressions 1 and 2 used a linear probability model. Because adherence behavior tends to be highly idiosyncratic and personalized, regression 2 added to regression 1 two individual-level variables. The first variable measured self-reported susceptibility to COVID-19 (observed in the first survey) and the second indicated whether adherence to preventive behavior is based on the respondent’s own decision or on that of anyone else in the household.

Regression 3 reran the model in regression 1 but using a probit model, assuming normally distributed disturbances. Rather than probit coefficients and for comparability with the results from regressions 1 and 2, the table reports marginal effects calculated at the variable means.

The magnitude of the coefficients is very similar across all three regressions, including across the linear probability and probit models.

The only significant individual-level variable was age in regression 1. Every 5 additional years of age were associated with a probability of wearing a mask that is lower by 1.5 percentage points. However, this variable was no longer significant when we controlled for self-reported susceptibility to COVID-19 and a variable measuring whether respondents made their own adherence decisions. Age was also no longer significant when the regression was estimated using a probit model rather than a linear probability model. No other individual-level variable was significant in any of the 3 regressions.

Of the household-level variables, geography was significantly associated with mask-wearing behavior: in all three regressions, respondents from the South were significantly more likely to wear a mask than those from the reference area (Akkar). Adherence was higher by close to 9 percentage points.

#### Sustained adherence to mask wearing

The results from regressions for sustained adherence to mask wearing are shown in Table 4. As in Table 3, regressions 1 and 2 used a linear probability model, with two additional variables in regression 2 on perceived susceptibility and decision making. Regression 3 shows the results of a probit model for sustained adherence.

**Table 4:** Sustained adherence to mask wearing.

	Regression 1		Regression 2		Regression 3	
	β	(S.E.)	β	(S.E.)	β	(S.E.)
	[95% CI]		[95% CI]		[95% CI]	
**Individual-level variables**						
Female	-0.01	(0.05)	-0.01	(0.05)	-0.01	(0.05)
	[-0.11:0.08]		[-0.11:0.08]		[-0.11:0.08]	
Married	-0.07	(0.09)	-0.06	(0.09)	-0.07	(0.09)
	[-0.25:0.11]		[-0.25:0.12]		[-0.24:0.10]	
Widowed	-0.05	(0.10)	-0.04	(0.10)	-0.06	(0.12)
	[-0.24:0.15]		[-0.24:0.16]		[-0.30:0.17]	
Age	-0.001	(0.003)	-0.001	(0.003)	-0.001	(0.003)
	[-0.006:0.004]		[-0.006:0.004]		[-0.006:0.004]	
Education						
Never attended school	Ref		Ref		Ref	
Elementary school	0.02	(0.04)	0.02	(0.04)	0.02	(0.04)
	[-0.06:0.10]		[-0.06:0.10]		[-0.06:0.10]	
Preparatory school	0.03	(0.05)	0.04	(0.05)	0.03	(0.05)
	[-0.06:0.13]		[-0.06:0.13]		[-0.06:0.12]	
Secondary school +	0.04	(0.06)	0.05	(0.06)	0.05	(0.06)
	[-0.07:0.16]		[-0.07:0.17]		[-0.06:0.17]	
Employed	0.07	(0.06)	0.07	(0.06)	0.07	(0.05)
	[-0.04:0.18]		[-0.04:0.18]		[-0.03:0.18]	
Job loss (due to COVID-19)	0.05	(0.06)	0.05	(0.06)	0.06	(0.05)
	[-0.06:0.16]		[-0.06:0.16]		[-0.04:0.17]	
Salary reduction (due to COVID-19)	-0.01	(0.05)	-0.01	(0.05)	-0.03	(0.06)
	[-0.12:0.09]		[-0.12:0.09]		[-0.15:0.09]	
Susceptible			-0.04	(0.04)		
			[-0.12:0.03]			
Own decision			-0.02	(0.03)		
			[-0.09:0.05]			
Smoker	0.01	(0.04)	0.01	(0.04)	0.01	(0.04)
	[-0.06:0.09]		[-0.06:0.09]		[-0.06:0.09]	
Chronic condition	-0.05	(0.03)	-0.05	(0.03)	-0.05	(0.03)
	[-0.12:0.02]		[-0.11:0.02]		[-0.12:0.01]	
Had COVID-19	0.08	(0.12)	0.09	(0.12)	0.09	(0.10)
	[-0.15:0.31]		[-0.14:0.32]		[-0.10:0.28]	
**Household-level variables**						
Household size	0.002	(0.004)	0.002	(0.004)	0.003	(0.004)
	[-0.01:0.01]		[-0.01:0.01]		[-0.06:0.11]	
Assets	0.003	(0.01)	0.005	(0.01)	0.002	(0.04)
	[-0.02:0.03]		[-0.02:0.03]		[-0.1:0.05]	
Severely food insecure	-0.02	(0.03)	-0.02	(0.03)	-0.02	(0.03)
	[-0.09:0.04]		[-0.09:0.04]		[-0.09:0.04]	
Cash assistance	-0.03	(0.04)	-0.03	(0.03)	-0.02	(0.04)
	[-0.10:0.05]		[-0.08:0.05]		[-0.10:0.05]	
ITS	-0.16**	(0.05)	-0.16**	(0.05)	-0.16**	(0.05)
	[-0.25:-0.07]		[-0.25:-0.07]		[-0.24:-0.07]	
Moved to ITS	0.02	(0.05)	0.02	(0.05)	0.01	(0.05)
	[-0.08:0.13]		[-0.08:0.13]		[-0.08:0.11]	
Moved from ITS	0.06	(0.06)	0.06	(0.06)	0.09	(0.05)
	[-0.05:0.17]		[-0.05:0.18]		[-0.02:0.20]	
**Governorate**						
Akkar	Ref		Ref		Ref	
Baalbeck-Hermel	0.03	(0.06)	0.03	(0.06)	0.03	(0.05)
	[-0.09:0.15]		[-0.08:0.15]		[-0.01:0.18]	
Beirut	0.27	(0.31)	0.25	(0.31)		
	[-0.33:0.87]		[-0.35:0.86]			
Beqaa	0.09+	(0.05)	0.09+	(0.05)	0.09+	(0.05)
	[-0.01:0.18]		[-0.01:0.19]		[-0.006:0.18]	
Mount Lebanon	0.09	(0.16)	0.11	(0.16)	0.10	(0.12)
	[-0.21:0.40]		[-0.20:0.42]		[-0.14:0.34]	
Nabatieh	0.04	(0.09)	0.03	(0.09)	0.03	(0.08)
	[-0.14:0.21]		[-0.14:0.21]		[-0.13:0.19]	
North	0.05	(0.06)	0.05	(0.06)	0.05	(0.05)
	[-0.07:0.17]		[-0.06:0.17]		[-0.06:0.16]	
South	0.06	(0.07)	0.06	(0.07)	0.07	(0.06)
	[-0.07:0.19]		[-0.07:0.19]		[-0.05:0.18]	
	**R** ^**2**^	0.09	**R** ^**2**^	0.10	**Pseudo- R** ^**2**^	0.09
	**F(43, 687)**	1.70	**F(45, 685)**	1.67	**Chi2(42)**	76.18
	**Observations**	731		731		729

+ p<0.1

* p<0.05

** p<0.01. All regressions include fixed effects for the week of data collection and duration between interviews.

As with the results in Table 3, the findings for sustained adherence were similar across the three regressions shown in Table 4, both in terms of magnitudes and significance of coefficients. The results suggest that none of the individual-level variables showed a significant association with sustained mask wearing.

One household-level variable that was found to be consistently significantly associated with continued mask-wearing was type of residence, with the probability of continued adherence 16 percentage points lower for ITS dwellers. However, we found no evidence that the probability of sustained adherence was significantly different for households moving into or out of an ITS over the 6 months. Other household level characteristics, including the household’s asset ownership, its food security status, its receipt of cash assistance showed no significant association with a respondent maintaining mask wearing.

Residents of the Bekaa showed a higher probability of sustained adherence than residence of the Akkar region, by 9 percentage point. But this result was only significant at the 10% level.

### Results for social gatherings

#### Avoidance of social gatherings

Table 5 reports the results of regressions of avoiding social gatherings.

**Table 5:** Avoiding social gatherings.

	Regression 1		Regression 2		Regression 3	
	β	(S.E.)	β	(S.E.)	β	(S.E.)
	[95% CI]		[95% CI]		[95% CI]	
**Individual-level variables**						
Female	0.01	(0.02)	0.02	(0.02)	0.01	(0.02)
	[-0.02:0.06]		[-0.02:0.06]		[-0.02:0.05]	
Married	0.05	(0.04)	0.05	(0.04)	0.06	(0.05)
	[-0.03:0.13]		[-0.03:0.13]		[-0.04:0.16]	
Widowed	0.03	(0.04)	0.03	(0.04)	0.03	(0.03)
	[-0.05:0.11]		[-0.06:0.11]		[-0.03:0.09]	
Age	0.0001	(0.001)	0.0001	(0.001)	0.0001	(0.001)
	[-0.002:0.002]		[-0.002:0.002]		[-0.002:0.002]	
Education						
Never attended school	Ref		Ref		Ref	
Elementary school	-0.02	(0.02)	-0.02	(0.02)	-0.03	(0.02)
	[-0.06:0.01]		[-0.06:0.01]		[-0.07:0.10]	
Preparatory school	-0.05**	(0.02)	-0.05**	(0.02)	-0.06**	(0.03)
	[-0.09:-0.01]		[-0.1:-0.01]		[-0.11:-0.01]	
Secondary school +	-0.07**	(0.02)	-0.08**	(0.02)	-0.09**	(0.04)
	[-0.12:-0.02]		[-0.13:-0.03]		[-0.17:-0.02]	
Employed	-0.03	(0.02)	-0.03	(0.02)	-0.03	(0.02)
	[-0.07:0.01]		[-0.07:0.02]		[-0.07:0.01]	
Susceptible			0.02	(0.01)		
			[-0.01:0.05]			
Own decision			-0.005	(0.01)		
			[-0.03:0.02]			
Smoker	0.004	(0.02)	0.005	(0.02)	0.005	(0.02)
	[-0.03:0.04]		[-0.03:0.04]		[-0.03:0.04]	
Chronic condition	-0.002	(0.01)	-0.003	(0.01)	-0.002	(0.01)
	[-0.03:0.02]		[-0.03:0.03]		[-0.03:0.02]	
**Household-level variables**						
Household size	-0.00003	(0.002)	-9e-7	(0.005)	0.0002	(0.002)
	[-0.003:0.003]		[-0.003:0.003]		[-0.003:0.004]	
Assets	-0.001	(0.005)	-0.001	(0.005)	-0.001	(0.005)
	[-0.01:0.01]		[-0.01:0.01]		[-0.04:0.02]	
Food insecure	0.03	(0.02)	0.03	(0.02)	0.03	(0.03)
	[-0.02:0.08]		[-0.02:0.08]		[-0.02:0.08]	
Cash assistance	-0.01	(0.01)	-0.01	(0.02)	-0.01	(0.01)
	[-0.04:0.02]		[-0.04:0.02]		[-0.04:0.02]	
ITS	-0.04*	(0.01)	-0.04*	(0.01)	-0.04*	(0.02)
	[-0.07:-0.01]		[-0.07:-0.01]		[-0.07:-0.01]	
**Governorate**						
Akkar	Ref		Ref		Ref	
Baalbeck-Hermel	0.0002	(0.03)	0.001	(0.03)	0.002	(0.02)
	[-0.05:0.05]		[-0.05:0.05]		[-0.05:0.05]	
Beirut	0.07	(0.07)	0.06	(0.07)		
	[-0.06:0.20]		[-0.07:0.2]			
Beqaa	0.01	(0.02)	0.01	(0.02)	0.01	(0.02)
	[-0.03:0.05]		[-0.03:0.05]		[-0.03:0.04]	
Mount Lebanon	0.09	(0.10)	0.09	(0.10)		
	[-0.12:0.29]		[-0.12:0.29]			
Nabatieh	0.01	(0.03)	0.005	(0.03)	0.005	(0.03)
	[-0.06:0.07]		[-0.07:0.07]		[-0.06:0.07]	
North	0.01	(0.02)	0.01	(0.02)	0.01	(0.02)
	[-0.03:0.07]		[-0.03:0.06]		[-0.03:0.05]	
South	0.02	(0.03)	0.02	(0.03)	0.02	(0.02)
	[-0.03:0.07]		[-0.03:0.07]		[-0.02:0.06]	
	**R** ^**2**^	0.03	**R** ^**2**^	0.03	**Pseudo- R** ^**2**^	0.05
	**F(34, 1536)**	1.31	**F(36, 1571)**	1.27	**Chi2(32)**	41.72
	**Observations**	1571		1571		1546

+ p<0.1

* p<0.05

** p<0.01. All regressions include fixed effects for the week of data collection.

As in the previous 2 tables, regressions 1 and 2 used a linear probability model, and regression 2 re-estimated the linear probability model with two additional individual-level variables, self-reported susceptibility to COVID-19, and a variable indicating whether adherence to preventive behavior was based on the respondent’s own decision or on that of anyone else in the household.

As in Table 3, regression 3 reran regression 1 using a probit model. The table reports marginal effects, calculated at the variable means.

The results from all three regressions suggested that unlike for mask wearing, among older Syrian refugees, education was significantly associated with a lower probability of avoiding social gatherings. Respondents with preparatory-level, secondary or higher education were significantly less likely to report avoiding social gatherings such as weddings and funerals than were respondents with no education by 5–6 percentage points and 7–8 percentage points, respectively.

The only other significant association with avoidance of social gatherings was residence in ITS. Across all three regressions, ITS residence was associated with a significantly lower probability of avoiding social gatherings by 4 percentage points.

#### Sustained avoidance of social gatherings

The results from regressions for sustained avoidance of social gatherings are shown in Table 6. Regressions 1, 2 and 3 are similar to those in Table 4, but with continued avoidance of social gathering over 6 months as the dependent variable.

**Table 6:** Sustained avoidance of social gatherings.

	Regression 1		Regression 2		Regression 3	
	β	(S.E.)	β	(S.E.)	β	(S.E.)
	[95% CI]		[95% CI]		[95% CI]	
**Individual-level variables**						
Female	-0.003	(0.03)	-0.004	(0.03)	-0.002	(0.02)
	[-0.05:0.05]		[-0.05:0.05]		[-0.05:0.05]	
Married	-0.03	(0.05)	-0.03	(0.05)	-0.03	(0.05)
	[-0.13:0.07]		[-0.13:0.07]		[-0.12:0.06]	
Widowed	-0.03	(0.05)	-0.03	(0.05)	-0.04	(0.07)
	[-0.14:0.08]		[-0.14:0.08]		[-0.18:0.10]	
Age	0.001	(0.001)	-0.001	(0.001)	0.001	(0.001)
	[-0.002:0.003]		[-0.002:0.003]		[-0.002:0.003]	
Education						
Never attended school	Ref		Ref		Ref	
Elementary school	0.0003	(0.02)	0.0006	(0.02)	0.002	(0.02)
	[-0.04:0.04]		[-0.04:0.04]		[-0.04:0.04]	
Preparatory school	-0.02	(0.03)	-0.02	(0.03)	-0.03	(0.03)
	[-0.07:0.03]		[-0.07:0.03]		[-0.08:0.03]	
Secondary school +	-0.03	(0.03)	-0.03	(0.03)	-0.04	(0.04)
	[-0.10:0.03]		[-0.1:0.03]		[-0.11:0.03]	
Employed	0.004	(0.03)	0.004	(0.03)	0.001	(0.03)
	[-0.06:0.06]		[-0.06:0.06]		[-0.05:0.06]	
Job loss (due to COVID-19)	-0.01	(0.03)	-0.01	(0.03)	-0.006	(0.03)
	[-0.07:0.05]		[-0.07:0.05]		[-0.07:0.05]	
Salary reduction (due to COVID-19)	-0.04	(0.03)	-0.03	(0.03)	-0.04	(0.03)
	[-0.10:0.03]		[-0.10:0.03]		[-0.11:0.03]	
Susceptible			-0.01	(0.02)		
			[-0.05:0.03]			
Own decision			-0.004	(0.02)		
			[-0.04:0.03]			
Smoker	-0.01	(0.02)	-0.01	(0.02)	-0.01	(0.02)
	[-0.05:0.03]		[-0.05:0.03]		[-0.05:0.02]	
Chronic condition	0.004	(0.02)	0.005	(0.02)	0.001	(0.02)
	[-0.03:0.04]		[-0.03:0.04]		[-0.03:0.04]	
Had COVID-19	-0.01	(0.05)	-0.01	(0.05)	-0.01	(0.05)
	[-0.12:0.09]		[-0.11:0.09]		[-0.11:0.09]	
**Household-level variables**						
Household size	-0.0005	(0.002)	-0.0006	(0.002)	-0.0003	(0.002)
	[-0.005:0.004]		[-0.005:0.003]		[-0.004:0.004]	
Assets	0.005	(0.007)	0.005	(0.01)	0.005	(0.006)
	[-0.01:0.02]		[-0.01:0.02]		[-0.01:0.02]	
Severely food insecure	0.01	(0.02)	0.01	(0.02)	0.01	(0.02)
	[-0.02:0.05]		[-0.02:0.05]		[-0.03:0.04]	
Cash assistance	0.01	(0.02)	0.01	(0.02)	0.01	(0.02)
	[-0.03:0.05]		[-0.03:0.05]		[-0.03:0.05]	
ITS	-0.04+	(0.02)	-0.04+	(0.02)	-0.04+	(0.02)
	[-0.09:-0.01]		[-0.08:0.01]		[-0.08:0.01]	
Moved to ITS	0.01	(0.03)	0.01	(0.03)	0.005	(0.03)
	[-0.05:0.07]		[-0.05:0.07]		[-0.05:0.06]	
Moved from ITS	0.002	(0.03)	0.002	(0.03)	-0.003	(0.03)
	[-0.06:0.06]		[-0.06:0.06]		[-0.06:0.05]	
**Governorate**						
Akkar	Ref		Ref		Ref	
Baalbeck-Hermel	0.02	(0.03)	0.02	(0.03)	0.02	(0.03)
	[-0.04:0.09]		[-0.04:0.09]		[-0.03:0.07]	
Beirut	0.15	(0.16)	0.14	(0.16)		
	[-0.16:0.45]		[-0.17:0.45]			
Beqaa	0.02	(0.03)	0.02	(0.03)	0.02	(0.02)
	[-0.03:0.07]		[-0.03:0.08]		[-0.03:0.06]	
Mount Lebanon	0.07	(0.09)	0.07	(0.09)	0.05	(0.05)
	[-0.1:0.24]		[-0.10:0.24]		[-0.04:0.15]	
Nabatieh	0.10*	(0.04)	0.10*	(0.04)	0.07*	(0.02)
	[0.01:0.19]		[0.01:0.19]		[0.03:0.11]	
North	0.08**	(0.03)	0.08**	(0.03)	0.07**	(0.02)
	[0.02:0.14]		[0.02:0.14]		[0.03:0.10]	
South	0.11**	(0.03)	0.11**	(0.03)	0.08**	(0.02)
	[0.04:0.17]		[0.04:0.17]		[0.05:0.11]	
	**R** ^**2**^	0.03	**R** ^**2**^	0.03	**Pseudo- R** ^**2**^	0.05
	**F(43, 1245)**	1.04	**F(45, 1243)**	1.00	**Chi2(42)**	48.57
	**Observations**	1289		1289		1285

+ p<0.1

* p<0.05

** p<0.01. All regressions include fixed effects for the week of data collection and duration between interviews.

Unlike for avoiding social gatherings, education was not significantly associated with continued avoidance, nor was any other individual-level variable.

ITS residents were shown to be less likely to maintain avoidance of social gatherings by 4 percentage points, a marginally (at the 10% level) significant difference. We found no such difference for movers into or out of ITSs over this 6-month period.

Geographical area of residence was significantly associated with the probability of continued adherence, with the districts of North Lebanon, South Lebanon and Nabatieh significantly more likely to maintain adherence than the district of Akkar, by 7–11 percentage points.

## Discussion

This project aimed to identify the main factors associated with adherence to mask wearing and social distancing among older Syrian refugees in Lebanon. It also investigated the factors correlated with sustained adherence to these two preventive measures over a six-month period. The findings suggest that few individual-level characteristics are significantly associated with adherence or continued adherence to wearing masks and avoiding social gatherings. The one exception is the negative associations between age and the probability of mask wearing, and education and the probability of avoiding social gatherings. The consistent correlates are structural than individual-level variables: residency in an ITS and residency in the poorest district (Akkar) appear to be significantly associated with poor adherence.

Regressions of adherence behavior used data collected in the first wave of the survey, spanning October-December of 2020. During this period, new cases of COVID-19 in Lebanon rose from close to 1000 per day in early October to 2000 per day in late December, while daily deaths during the same period went from 6 to 16 [34].

Regressions of sustained adherence used data collected in February-May of 2021. February of 2021 is the tail end of Lebanon’s second COVID-19 wave, with new daily cases close to 3000 in early February, dropping to around 400 by mid-May. Daily deaths peak at around 115 in early February and decrease to around 20 by mid-May [34]. The first doses of the vaccine were administered in late February, but the rollout remained slow with the total doses administered still under 600,000 by mid-May, which put the share of the 16 or older population that received one dose of vaccine by then at under 10% [35].

Although the literature reports a gender difference in mask wearing [36–39], we found no difference between males and females in mask wearing or sustained mask wearing among older Syrian refugees. The literature sometimes explains the lower adherence of men by the fact that they are more likely than women to perceive wearing masks as a sign of weakness. But one other study of the Middle East and North Africa shows that perception of masks tends to be lowest in Lebanon [40].

Previous studies have shown that over the entire age range of the adult population, mask wearing tends to increase with age [22, 39]. One study focusing only on older adults found no significant effect of age on acceptance of preventive measures and adherence to them [41]. We find in regression 1 that among elderly Syrian refugees, the older are significantly less likely to wear a mask, with the probability of wearing a mask 1.5 percentage points lower for every 5 additional years of age. However, we do not attach a lot of confidence to this result, as the significance of the finding is lost when the regression is estimated using a probit model.

Findings from the literature suggest that education is associated with higher likelihood of wearing a mask [22, 42, 43], however, we found no such difference in mask wearing by education level. Where we did find a significant association with education was in avoiding social gatherings. Respondents with elementary education or less were likelier to comply (and to continue complying) than respondents with preparatory education or more. These partial correlations controlled for labor market status and socioeconomic standing. This contrasts with findings from Germany [44], but is in line with results from a survey of adults from North America and Europe [45].

A finding of concern is a lack of statistical difference in adherence for the relatively more vulnerable (respondents with chronic conditions and older respondents). There are similar results in the literature reported, both in Ethiopia [46], and among older adults in Thailand [47]. There may be several potential explanations for this finding. One explanation is that older Syrian refugees already had significant difficulty in accessing healthcare, even before the economic crisis in Lebanon and the pandemic and showed poorer mental health and more cognitive difficulties than younger refugees [48–50]. Another factor could be that people with chronic conditions were likely to exhibit significantly worse mental health during the pandemic [51], and mental health affects people’s willingness and ability to adhere to preventive measures.

Variables that were significantly associated with different adherence behaviors were more structural and environmental. There were significant differences in adherence to both preventive practices by district of residence. Residents of the South were significantly more likely to mask, residents of the Bekaa to continue masking, than were residents of Akkar. Respondents in the South, North and Nabatieh were also significantly more likely to maintain avoidance of social gatherings than were respondents from Akkar. This is consistent with findings from an earlier nationally representative survey that found lower levels of knowledge and practice on COVID-19 prevention in Akkar, even among Lebanese residents [52].

Qualitative studies have documented the material, psychological, economic, and socio-cultural difficulty of distancing among Syrian refugees [53, 54]. However, we found that even among Syrian refugees, and after controlling for socio-economic status, residents of ITSs showed a discretely lower probability of adherence to all but one preventive measure than did other Syrian refugees. Except for mask wearing, residents of ITSs were significantly less likely to report adherence to the other three preventive practices we were tracking, regardless of the statistical approach used or the control variables included in the regression. The difference in compliance was both significant and large in magnitude. The lack of statistical distinction between refugees living in and out of ITSs in mask wearing was perhaps the result of the distribution of hygiene kits (including masks) that took place in the ITS camps during the first few months of the pandemic [17, 54]. However, with the rapidly deteriorating economic situation in the country, even a low cost NPI such as masking can become unaffordable if refugees are expected to secure their own masks.

The lower rates of adherence in ITS should be of major concern for the control of the spread of COVID-19, as well as for emergency preparedness, safety, and public health more generally, as the more recent outbreak of cholera in Lebanon goes to show [55]. Indeed, ITSs are built using temporary materials and offer very limited thermal or environmental protection to residents. The quality of water, sanitation, and hygiene is typically extremely poor with exposed latrines, open sewage, limited access to clean water, and no wastewater management. Residents are exposed to the risk of flooding, poor air quality, and water-borne diseases [56]. Furthermore, ITSs are densely populated, severely limiting the possibility of quarantine, isolation, and distancing [57].

With the baseline conditions in ITSs as exposed to public health hazards, the serious challenge residents face to distancing and avoiding social gatherings should be taken into account in designing emergency public health policies for disease containment in such a context. Our findings corroborate in behavior what previous research has documented about the physical and economic difficulty of distancing.

### Limitations

The findings should be read with several limitations in mind: the sample is representative of refugee households with senior members who are among the beneficiaries of NRC, and not of the larger population of Syrian refugees in Lebanon. The data used to generate the results were self-reported so they are prone to all the bias that self-reported data may involve. The survey was conducted over the phone with no opportunity for the data collector to verify some of the responses related to dwelling characteristics and asset ownership. In either choice of model (linear probability or probit), we are making strong assumptions about the functional form of the relationship between the dependent and explanatory variables. The partial correlations estimated in the main regressions are linear by construction, because of our choice of a linear probability model. We also looked at each preventive practice separately and did not look at concurrent behaviors.

## Conclusion

Our findings generally suggest that within the vulnerable subgroup of older Syrian refugees, few individual-level variables are associated with differences in adherence (and continued adherence) to preventive measures, especially the habituating practice of mask wearing. Instead, variation in adherence seems to be related to structural or environmental factors, such as geographic district of residence or residence in an ITS. The fact that compliance with one preventive practice (mask wearing) was shown to be possible in ITSs when masks are made available holds lessons for policy making: tailoring interventions to the local conditions and constraints that ITS residents endure can be effective at protecting public health without imposing undue costs on refugees or issuing directives that are exceedingly difficult for ITS residents to comply with.

Determining the correlates of adherence to NPIs helps in identifying subgroups whose behavior has been riskier or whose context makes compliance disproportionately costly. Even with the WHO declaring the end of the pandemic in May of 2023, the findings could inform the design of interventions for prevention, preparedness, and management of future emergencies, particularly in the context of an unraveling economy like Lebanon’s. Identifying variables associated with continued adherence helps update and refine interventions in the face of protracted crises in the future.

## References

[1] Isma’eel H., El Jamal N., Yazbik Dumit N., & Al-Chaer E. (2020). Saving the Suffering Lebanese Healthcare Sector: Immediate Relief while Planning Reforms. *Arab Reform Initiative*.

[2] MSF. (2021). *Healthcare system in Lebanon disintegrates as political vacuum persists*. https://www.msf.org/healthcare-system-lebanon-crumbles-amidst-political-and-economic-crisis

[3] Shallal A., Lahoud C., Zervos M., & Matar M. (2021). Lebanon is losing its front line. *J Glob Health*, 11, 03052. doi: 10.7189/jogh.11.03052 33828836 PMC8005300

[4] Youssef D., Issa O., Kanso M., Youssef J., Abou-Abbas L., & Abboud E. (2022). Practice of non-pharmaceutical interventions against COVID-19 and reduction of the risk of influenza-like illness: a cross-sectional population-based study. *Journal of Pharmaceutical Policy and Practice*, doi: 10.1186/s40545-022-00450-y 36042506 PMC9424835

[5] Makhoul J., Kabakian-Kasholian T., & Chaiban L. Analyzing the social context of health information and misinformation during the COVID-19 pandemic: a case of emerging inequities in Lebanon. *Global Health Promotion*, 2021, March; 28(1): 33–41 doi: 10.1177/1757975920984178 33472532 PMC7820514

[6] Koweyes J., Salloum T., Haidar S., Merhi G., & Tokajian S. COVID-19 Pandemic in Lebanon: One Year Later, What Have We Learnt? *mSystems*, 2021, Apr;6(2). 10.1128/mSystems.00351-21 PMC8546977 33879497

[7] Cheng VC, Wong SC, Chuang VW, So SY, Chen JH, Sridhar S, et al. The role of community-wide wearing of face mask for control of coronavirus disease 2019 (COVID-19) epidemic due to SARS-CoV-2. *J Infect*. 2020 Jul;81(1):107–114. doi: 10.1016/j.jinf.2020.04.024 32335167 PMC7177146

[8] Siedner M. J., Harling G., Reynolds Z., Gilbert R. F., Haneuse S., Venkataramani A. S., et al. (2020). Social distancing to slow the US COVID-19 epidemic: Longitudinal pretest-posttest comparison group study. *PLoS Med*, 17(8), e1003244. doi: 10.1371/journal.pmed.1003244 32780772 PMC7418951

[9] Teslya A., Pham T. M., Godijk N. G., Kretzschmar M. E., Bootsma M. C. J., & Rozhnova G. (2020). Impact of self-imposed prevention measures and short-term government-imposed social distancing on mitigating and delaying a COVID-19 epidemic: A modelling study. *PLoS Med*, 17(7), e1003166. doi: 10.1371/journal.pmed.1003166 32692736 PMC7373263

[10] Xiao Y., & Torok M. E. (2020). Taking the right measures to control COVID-19. *Lancet Infect Dis*, 20(5), 523–524. doi: 10.1016/S1473-3099(20)30152-3 32145766 PMC7128408

[11] WHO. (2020). *Pandemic fatigue*: *reinvigorating the public to prevent COVID-19*: *policy framework for supporting pandemic prevention and management*: *revised version November* 2020.

[12] Badre D. (2021, January 24). How We Can Deal with ‘Pandemic Fatigue’. *Scientific American*.

[13] Petherick A., Goldszmidt R., Andrade E. B., Furst R., Hale T., Pott A., et al. (2021). A worldwide assessment of changes in adherence to COVID-19 protective behaviours and hypothesized pandemic fatigue. *Nat Hum Behav*, 5(9), 1145–1160. doi: 10.1038/s41562-021-01181-x 34345009

[14] Dempster H., Ginn T., Graham J., Guerrero Ble M., Jayasinghe D., & Shorey B. (2020). Locked Down and Left Behind: The Impact of COVID-19 on Refugees’ Economic Inclusion. Washington DC: Center for Global Development and Refugees International.

[15] Chaaban J., Salti N., Ghattas H., Moussa W., Irani A., Jamaluddine Z., et al. (2020). Multi-purpose cash assistance in Lebanon: Impact evaluation on the well-being of Syrian refugees. *American University of Beirut Press*. *https://www.aub.edu.lb/fafs/agri/aedrg/Documents/AUB%20Impact%20Study_Final_print.pdf*.

[16] UNHCR, UNICEF, & WFP. (2019). VASyR 2019: Vulnerability Assessment of Syrian Refugees in Lebanon. UNHCR Beirut, Lebanon.

[17] Fouad F. M., McCall S. J., Ayoub H., Abu-Raddad L. J., & Mumtaz G. R. Vulnerability of Syrian refugees in Lebanon to COVID-19: quantitative insights. *Conflict and Health*, 2021;15(13), 1–6, doi: 10.1186/s13031-021-00349-6 33673855 PMC7934989

[18] Nikolich-Zugich J., Knox K., Tafich Rios C., Natt B., Bhattacharya D., & Fain M. SARS-CoV-2 and COVID-19 in older adults: what we may expect regarding pathogenesis, immune responses, and outcomes. *Geroscience*, 2020 Apr;42(2):505–514, doi: 10.1007/s11357-020-00186-0 32274617 PMC7145538

[19] Patel JA, Nielsen FBH, Badiani AA, Assi S, Unadkat VA, Patel B, et al. Poverty, inequality and COVID-19: the forgotten vulnerable. *Public Health*. 2020 Jun;183:110–111. doi: 10.1016/j.puhe.2020.05.006 32502699 PMC7221360

[20] Singhal S., Kumar P., Singh S. et al. Clinical features and outcomes of COVID-19 in older adults: a systematic review and meta-analysis. *BMC Geriatr* 2021 May 19;21(1):321, doi: 10.1186/s12877-021-02261-3 34011269 PMC8133052

[21] Adjodah D., Dinakar K., Chinazzi M., Fraiberger S. P., Pentland A., Bates S., et al. (2021). Association between COVID-19 outcomes and mask mandates, adherence, and attitudes. *PLoS One*, 16(6), e0252315. doi: 10.1371/journal.pone.0252315 34161332 PMC8221503

[22] Badillo-Goicoechea E, Chang TH, Kim E, LaRocca S, Morris K, Deng X, et al. Global trends and predictors of face mask usage during the COVID-19 pandemic. *BMC Public Health*. 2021 Nov 15;21(1):2099. doi: 10.1186/s12889-021-12175-9 34781917 PMC8667772

[23] Daoust J. F. Elderly people and responses to COVID-19 in 27 Countries. *PLOS One*, 2020.15(7), e0235590. doi: 10.1371/journal.pone.0235590 32614889 PMC7332014

[24] Domiati S., Itani M., & Itani G. Knowledge, Attitude, and Practice of the Lebanese Community Toward COVID-19. *Front Med* *(Lausanne)*, 2020, 7, 542. doi: 10.3389/fmed.2020.00542 33015096 PMC7461812

[25] Abou-Abbas L., Nasser Z., Fares Y., Chahrour M., El Haidari R., & Atoui R. (2020). Knowledge and practice of physicians during COVID-19 pandemic: a cross-sectional study in Lebanon. *BMC Public Health*, 20(1), 1474. 10.1186/s12889-020-09585-6 32993603 PMC7523262

[26] Nasser Z., Fares Y., Daoud R., & Abou-Abbas L. (2020). Assessment of knowledge and practice of dentists towards Coronavirus Disease (COVID-19): a cross-sectional survey from Lebanon. *BMC Oral Health*, 20(1), 281. doi: 10.1186/s12903-020-01273-6 33050914 PMC7552581

[27] Sakr S., Ghaddar A., Sheet I., Eid A. H., & Hamam B. Knowledge, attitude and practices related to COVID-19 among young Lebanese population. *BMC Public Health*, 2021. 21(1), 653. doi: 10.1186/s12889-021-10575-5 33823826 PMC8022301

[28] Melki J., Tamim H., Hadid D., Farhat S., Makki M., Ghandour L., et al. Media Exposure and Health Behavior during Pandemics: The Mediating Effect of Perceived Knowledge and Fear on Compliance with COVID-19 Prevention Measures. *Health Commun*, 2020, 1–11. doi: 10.1080/10410236.2020.1858564 33327785

[29] Chan EYY, Kim JH, Kwok KO, Huang Z, Hung KKC, Wong ELY, et al. Population Adherence to Infection Control Behaviors during Hong Kong’s First and Third COVID-19 Waves: A Serial Cross-Sectional Study. *Int J Environ Res Public Health*. 2021 Oct 24;18(21):11176. 34769694 10.3390/ijerph182111176 PMC8583559

[30] Makki F., Sedas P. S., Kontar J., Saleh N., & Krpan D. Compliance and stringency measures in response to COVID-19: A regional study. *Journal of Behavioral Economics for Policy*, 2020, 4(S2), 15–24.

[31] Jeste DV, Palmer BW, Appelbaum PS, et al. A New Brief Instrument for Assessing Decisional Capacity for Clinical Research. *Arch Gen Psychiatry*. 2007;64(8):966–974. doi: 10.1001/archpsyc.64.8.966 17679641

[32] Abdulrahim S., Ghattas H., McCall S., Sibai A., Chaaya M., Salti N., et al. Tracking adherence of older refugees to COVID-19 preventive measures in response to changing vulnerabilities: A multi-level, panel study to inform humanitarian response in Lebanon, 2021 [Survey Documentation]. Beirut, Lebanon, 2021, May 23.

[33] McCall S., El Khoury T., Salibi N., Abi Zeid B., El Haddad M., Alawieh M., et al. Development of a Prediction Model for the Management of Noncommunicable Diseases Among Older Syrian Refugees Amidst the COVID-19 Pandemic in Lebanon. *JAMA Netw Open*. 2022 Oct 3;5(10):e2231633. doi: 10.1001/jamanetworkopen.2022.31633 36227600 PMC9561955

[34] Dong E, Ratcliff J, Goyea TD, Katz A, Lau R, Ng TK, et al. The Johns Hopkins University Center for Systems Science and Engineering COVID-19 Dashboard: data collection process, challenges faced, and lessons learned. *Lancet Infect Dis*. 2022 Aug 31:S1473–3099(22)00434–0. 10.1016/S1473-3099(22)00434-0 PMC9432867 36057267

[35] Roser M., & Ortiz-Ospina E. *Total COVID vaccine doses administered*. Retrieved from Our World in Data: https://ourworldindata.org/, accessed on November 6, 2022.

[36] Cassino D., & Besen-Cassino Y. Of Masks and Men? Gender, Sex, and Protective Measures during COVID-19. *Politics & Gender*, 2020, 16(4), 1052–1062.

[37] Chuang Y., & Liu C. Who wears a mask? Gender differences in risk behaviors in the COVID-19 early days in Taiwan. *Economics Bulletin*, 2020, 40(4), 2619–2627.

[38] Haischer MH, Beilfuss R, Hart MR, Opielinski L, Wrucke D, Zirgaitis G, et al. Who is wearing a mask? Gender-, age-, and location-related differences during the COVID-19 pandemic. *PLoS One*. 2020 Oct 15;15(10):e0240785. doi: 10.1371/journal.pone.0240785 33057375 PMC7561164

[39] Okten I. O., Gollwitzer A., & Oettingen G. Gender differences in preventing the spread of coronavirus. *Behavioral Science and Policy*, 2020, 6(2), doi: 10.31234/osf.io/ch4jy

[40] Stephan R., Khurma M. & Chouhod Y. Attitudes towards mask wearing in MENA: the impact of gender and education. *Viewpoint Series*, Wilson Center, April 20, 2022, Washington D.C., https://www.wilsoncenter.org/article/attitudes-towards-mask-wearing-mena-impact-gender-and-education.

[41] Bearth A., Luschsinger L., & Siegrist M. (2021). Reactions of older Swiss adults to the COVID-19 pandemic: A longitudinal survey on the acceptance of and adherence to public health measures. *Social Science and Medicine*, 10.1016/j.socscimed.2021.114039. PMC8129997 34051558

[42] Abeya SG, Barkesa SB, Sadi CG, Gemeda DD, Muleta FY, Tolera AF, et al. Adherence to COVID-19 preventive measures and associated factors in Oromia regional state of Ethiopia. *PLoS One*. 2021 Oct 20;16(10), doi: 10.1371/journal.pone.0257373 34669723 PMC8528333

[43] Ditekemena JD, Nkamba DM, Muhindo HM, Siewe JNF, Luhata C, Van den Bergh R, et al. Factors associated with adherence to COVID-19 prevention measures in the Democratic Republic of the Congo (DRC): results of an online survey. *BMJ Open*. 2021 Jan 18;11(1), doi: 10.1136/bmjopen-2020-043356 33462101 PMC7813390

[44] Lüdecke D. and von dem Knesebeck O. Protective behavior in course of the COVID-19 outbreak–survey results from Germany. *Frontiers in Public Health*, 24 September 2020, doi: 10.3389/fpubh.2020.572561 33072712 PMC7543680

[45] Coroiu A, Moran C, Campbell T, Geller AC. Barriers and facilitators of adherence to social distancing recommendations during COVID-19 among a large international sample of adults. *PLoS One*. 2020 Oct 7;15(10) doi: 10.1371/journal.pone.0239795 33027281 PMC7540845

[46] Dires A, Gedamu S, Getachew Y. Perception of COVID-19 Prevention Methods Efficacy and Intention to Use Among Patients with Chronic Disease in Dessie Town, Northeast Ethiopia: A Multicentered Cross-sectional Study. *J Multidiscip Healthc*. 2021;14:1325–1339, doi: 10.2147/JMDH.S313796 34113120 PMC8186997

[47] Yodmai K, Pechrapa K, Kittipichai W, Charupoonpol P, Suksatan W. Factors Associated with Good COVID-19 Preventive Behaviors Among Older Adults in Urban Communities in Thailand. *Journal of Primary Care & Community Health*. 2021;12. doi: 10.1177/21501327211036251 34334008 PMC8326623

[48] Abi Chahine M, Kienzler H. Ageism, an invisible social determinant of health for older Syrian refugees in Lebanon: a service providers’ perspective. Confl Health. 2022 Nov 24;16(1):62. doi: 10.1186/s13031-022-00491-9 ; PMCID: PMC9694515. 36434728 PMC9694515

[49] Chemali Z, Borba CPC, Johnson K, Khair S, Fricchione GL. Needs assessment with elder Syrian refugees in Lebanon: Implications for services and interventions. Glob Public Health. 2018 Sep;13(9):1216–1228. doi: 10.1080/17441692.2017.1373838 28895503 PMC5847430

[50] Strong J, Varady C, Chahda N, Doocy S, Burnham G. Health status and health needs of older refugees from Syria in Lebanon. Confl Health. 2015 Apr 9;9:12. doi: 10.1186/s13031-014-0029-y ; PMCID: PMC4459463. 26056531 PMC4459463

[51] Salah H.; Ibrahim Rabie A.S.; Said A.S.A.; AlAhmad M.M.; Shaaban A.H.; Khalil D.M.; et al. COVID-19′s Psychological Impact on Chronic Disease Patients Seeking Medical Care. *Healthcare* 2023, 11, 888. doi: 10.3390/healthcare11060888 36981545 PMC10048099

[52] UNICEF. (2020). Knowledge, Attitudes and Practice Towards COVID-19 in Lebanon. Beirut: UNICEF.

[53] Al Munajed D. and Ekren E. Exploring the impact of multidimensional refugee vulnerability on distancing as a protective measure against COVID-19: the case of Syrian refugees in Lebanon and Turkey. *Journal of Migration and Health*, Volumes 1–2, doi: 10.1016/j.jmh.2020.100023 34405174 PMC8352139

[54] Moawad P. & Andres L. (2020). Tackling COVID-19 in informal tented settlements (Lebanon): an assessment of preparedness and response plans and their impacts on the health vulnerabilities of Syrian refugees in Lebanon. *Journal of Migration and Health*, Volumes 1–2, doi: 10.1016/j.jmh.2020.100011 34405166 PMC8351976

[55] News UN 2022. Cholera spreading throughout Lebanon, WHO warns. November 1, 2022. Retrieved on November 9, 2022 at https://news.un.org/en/story/2022/11/1130067.

[56] Kassem I. and Jaafar K. 2020. The potential impact of water quality on the spread and control of COVID-19 in Syrian refugee camps in Lebanon. *Water International*, 45(5) 2020, 423–429.

[57] Moawad P., & Andres L. (2020). Decoding Syrian refugees’ COVID-19 vulnerability in informal tented settlements: a community-/refugee-led approach to mitigate a pandemic outbreak. *The Town Planning Review*, 92(1), 33–9.

